# Case Report: Report of Two Cases of Interstitial Lung Disease Caused by Novel Compound Heterozygous Variants in the *ABCA3* Gene

**DOI:** 10.3389/fgene.2022.875015

**Published:** 2022-04-06

**Authors:** Fang Chen, Zhiwei Xie, Victor Wei Zhang, Chen Chen, Huifeng Fan, Dongwei Zhang, Wenhui Jiang, Chunli Wang, Peiqiong Wu

**Affiliations:** ^1^ Respiratory Department of Guangzhou Women and Children’s Medical Center, Guangzhou, China; ^2^ Department of Human and Molecular Genetics, Baylor College of Medicine, Houston, TX, United States; ^3^ AmCare Genomics Lab, Guangzhou, China

**Keywords:** ABCA3, interstitial lung disease, ILD, surfactant protein dysfunction, pediatric

## Abstract

Interstitial lung disease (ILD) is a heterogeneous group of pulmonary disorders involving the lung interstitium and distal airways, also known as diffuse lung disease. The genetic defects resulting in alveolar surfactant protein dysfunction are a rare cause of ILD in pediatric patients. We report two unrelated pediatric patients with shortness of breath, dyspnea and hypoxemia, and the chest CT findings including patchy ground-glass opacity in both lung fields, suggestive of diffuse ILD. One patient was a full-term male infant who had shortness of breath a few hours after the birth, and then developed into severe respiratory distress syndrome (RDS). Whole exome sequencing revealed novel compound heterozygous variants in the *ABCA3* gene (NM_001,089.3): paternally inherited c.4035+5G > A and c.668T > C (p.M223T), and maternally inherited c.1285+4A > C. The second patient was a 34-month-old boy with onset of chronic repeated cough and hypoxemia at 9 months of age. We unveiled novel compound heterozygous *ABCA3* variants (c.704T > C, p.F235S; c.4037_4040del, p.T1346Nfs*15) in this patient. Surfactant protein dysfunction due to bi-allelic mutations in the *ABCA3* gene was the cause of ILD in two patients. The novel mutations found in this study expanded the spectrum of known mutations in the *ABCA3* gene.

## Introduction

Pediatric interstitial lung disease (ILD) is a group of rare diseases that occurs in the neonatal or infancy period, which are characterized by varying degrees of inflammation and fibrosis in the lung, and diffuse interstitial changes in imaging examinations. It is being increasingly recognized that ILD affecting newborns, infants and young children usually has an underlying genetic causes, which may be identified through genetic analysis based upon next-generation sequencing methods (Nogee, 2017; Bush et al., 2020; Nathan et al., 2020). Genetic mutations resulting in surfactant protein dysfunction have been regarded as an important etiology of full-term neonates with acute and progressive respiratory distress and chronic respiratory disease in older children. These symptoms are associated with significant morbidity and mortality (Turcu et al., 2013; Nogee, 2019).

Pulmonary surfactant is a complex of phospholipids (90%) and proteins (10%) by mass, which functions to keep alveoli from collapsing at expiration (Carreto-Binaghi et al., 2016). There are four surfactant proteins have been identified, including surfactant proteins A (SP-A), B (SP-B), C (SP-C), and D (SP-D). Other proteins such as ATP binding cassette number A3 (ABCA3) and thyroid transcription factor-1 (TTF1) are required for the normal structure and functions of pulmonary surfactants. Research shows that the genetic mutations in the SP-B gene (*SFTPB*), SP-C gene (*SFTPC*), *ABCA3* and *TTF1* genes have been identified as the main causes of surfactant protein dysfunction (Turcu et al., 2013; Nathan et al., 2020). ABCA3 protein is a member of the highly conserved multispan transmembrane ABC superfamily of proteins that hydrolyze ATP to move substrates across biological membranes, which mediates the transport of choline phospholipids and is required for lamellar body biogenesis (Peca et al., 2015). The clinical manifestations of pulmonary diseases caused by *ABCA3* gene mutations vary greatly, ranging from neonatal respiratory failure to childhood or adult diffuse ILD (Panigrahy et al., 2014; Klay et al., 2020; Al-Iede et al., 2021). Here, we report two unrelated pediatric patients with interstitial lung disease caused by compound heterozygous mutations in the *ABCA3* gene.

## Case Presentation

### Patients

Two patients were referred to our department of pediatric respiratory for pneumonia without previous clinical diagnosis. This study was approved by the ethical committees of Guangzhou Women and Children’s Medical Center. Written informed consent was obtained from the parents. Clinical characteristics of the two patients were summarized in Table 1.

**TABLE 1:** Patient characteristics.

Characteristics	Patient 1	Patient 2
Sex	Male	Male
Age at onset	A few hours after birth	9 months
Gestational age	41 weeks	39 weeks
Pulmonary presentation	Severe RDS; ILD; Persistent tachypnea and hypoxemia	ILD; Hypoxemia
Extrapulmonary symptoms	Poor weight gain	Poor weight gain; Developmental delay; Pestle finger
Family history	The elder brother suffered from repeated pneumonia after birth, and died of severe pneumonia when he was 7 months old	None
Age at genetic diagnosis	70 days	34 months
*ABCA3* gene mutations	c.4035+5G > A, c.668T > C (p.M223T)	c.704T > C (p.F235S)
	c.1285+4A > C	c.4037_4040del (p.T1346Nfs*15)
ABCA3 deficiency treatment regimen	Azithromycin	Azithromycin
	Hydroxychloroquine	Budesonide suspension
	Dexamethasone	Ipratropium bromide solution
Additional therapies	Ventilator support	Oxygen therapy
Maximal respiratory support	CPAP	Medium flow oxygen supply
Current outcome	Died aged 94 days	Alive, 41-month-old without respiratory support

RDS, respiratory distress syndrome; ILD, interstitial lung disease; CPAP, continuous positive airway pressure.

#### Patient 1

**FIGURE 1:**
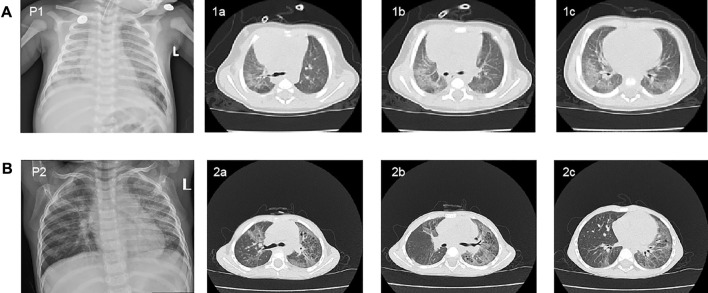
Chest X-ray and high-resolution computed tomography (HRCT) scan findings in two patients. **(A)** Chest X-ray revealed multiple patchy shadows in both lung fields of patient 1 at 60 days of age (P1). The chest HRCT of the patient showed large scattered ground-glass opacity in both lung fields at 46 days of age (1a, 1b, and 1c). **(B)** Chest X-ray of patient2 revealed the bilateral diffuse plaque-like shadows (P2). The chest HRCT scan showed multiple striped fuzzy shadows and ground-glass opacity in both lung fields (2a, 2b, and 2c).

Patient 1 was a 46-day-old male infant, who presented with repeated shortness of breath and cough. The infant was born full term at 41 weeks of gestation with a birth weight of 3,660 g. He had shortness of breath without an obvious organic cause on the first day after birth and then he had a single cough 1 week later that developed into paroxysmal continuous cough, accompanied by shortness of breath and sputum. Clinical symptoms of the infant were not alleviated after treatments in local hospitals several times. The patient was admitted to our hospital because of the aggravated cough and shortness of breath. Chest X-ray of the patient revealed multiple patchy shadows in both lung fields (Figure 1A). The chest high-resolution computed tomography (HRCT) showed large scattered ground-glass opacity in both lung fields (Figures 1A, 1a–c). He was the second child of non-consanguineous parents. In his family history, his older brother had a history of repeated pneumonia after birth, and died of severe pneumonia when he was 7 months old.

**FIGURE 2:**
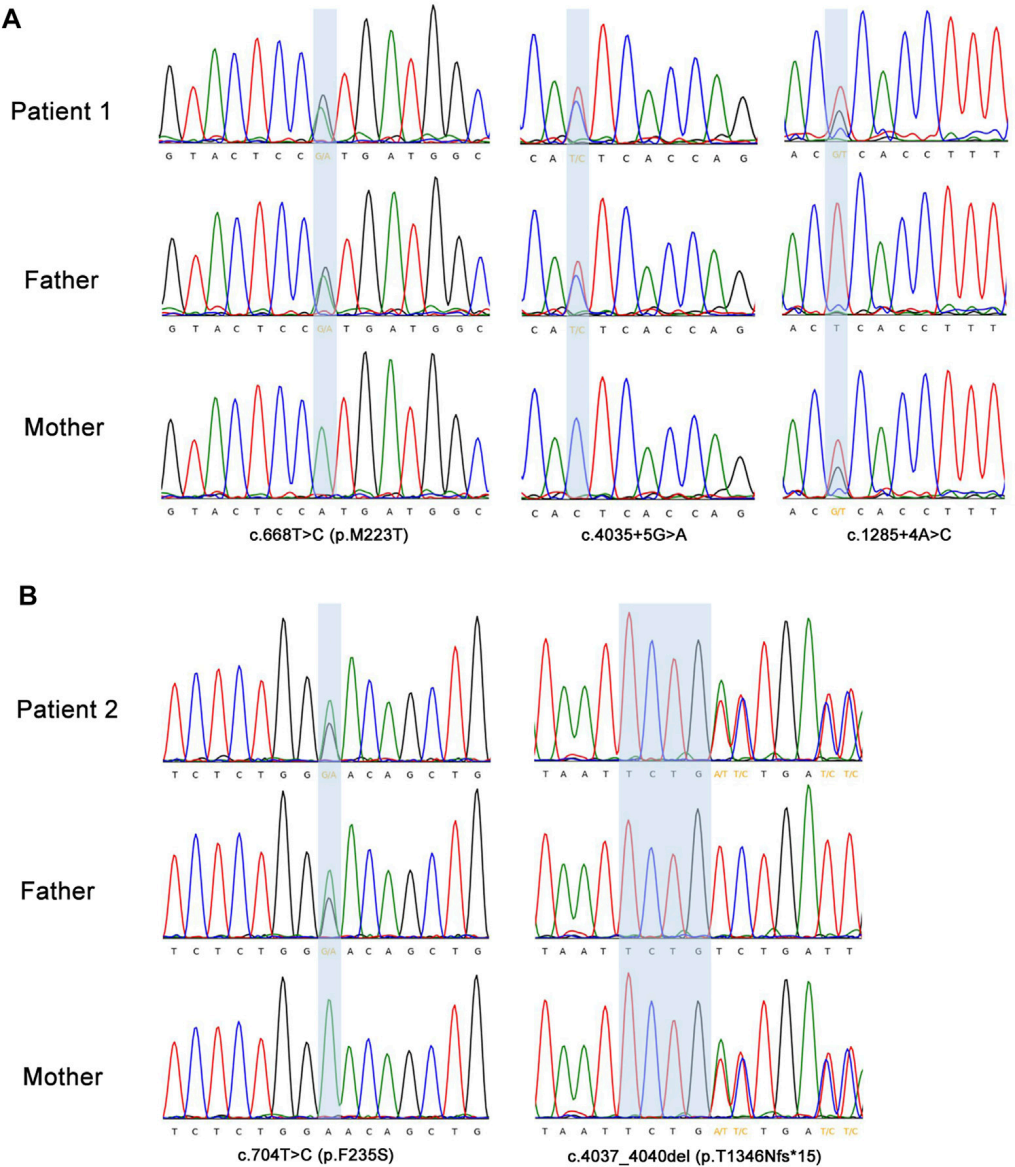
Sanger sequencing confirmation of the *ABCA3* gene variants detected by exome sequencing. **(A)** The highlighting blue background area showed two heterozygous paternally inherited variants (c.4035+5G > A; c.668T > C, p.M223T) and a heterozygous maternally inherited variant (c.1285+4A > C) in *ABCA3* gene in patient 1. **(B)** Patient 2 had a heterozygous missense variant c.704T > C (p.F235S) inherited from the father and a heterozygous frameshift variant c.4037_4040del (p.T1346Nfs*15) from the mother in the *ABCA3* gene. Sanger sequencing showed the antisense sequence alignment of the *ABCA3* gene.

**FIGURE 3:**
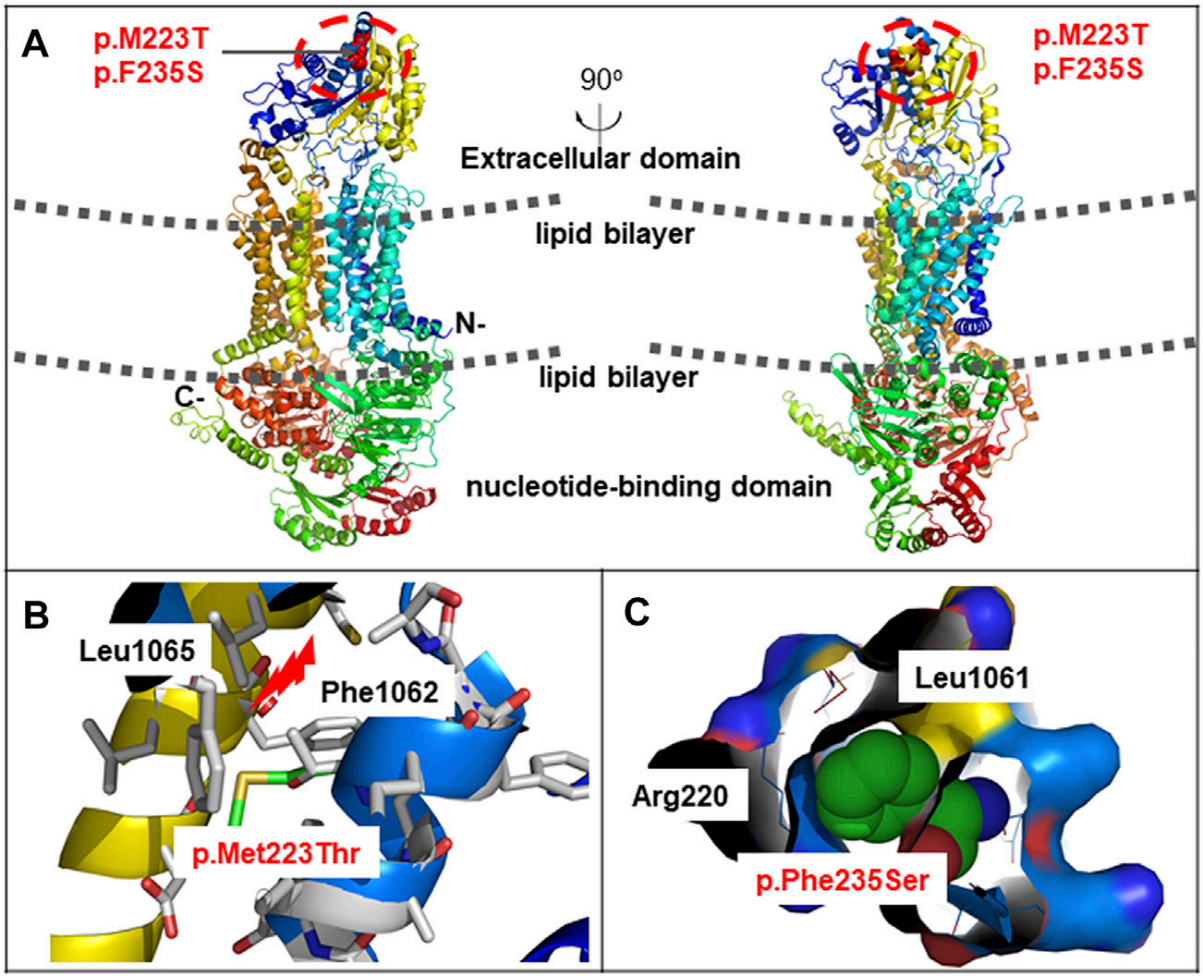
Schematic representation of missense variants p.M223T and p. F235S on the ABCA3 protein. **(A)** Predicted tertiary structure of the ABCA3 transporter by PyMOL. ABCA3 is an integral membrane protein that contains 12 membrane-spanning helices and two cytosolic nucleotide binding domains. The two missense variants were located on the extracellular domain. **(B)** Spatial disposition of the amino acid residues Met223, Leu1065, and Phe1062. **(C)** Spatial disposition of the amino acid residues Phe235, Arg220, and Leu1061.

On day 70 of life, whole exome sequencing revealed compound heterozygous variants in the *ABCA3* gene (NM_001,089); paternally inherited c.4035+5G > A in intron 26 and c.668T > C (p.M223T) in exon 8, and maternally inherited c.1285+4A > C in intron 11. Sanger sequencing was performed to verity the three variants in the patient and his parents (Figure 2A). The three variants of the *ABCA3* gene were all absent from the gnomAD and ExAC databases, and have not been reported in the literature. The intron variants c.4035+5G > A and c.1285+4A > C were predicted to affect RNA splicing by several different splice site algorithms (SpliceSiteFinder-like, MaxEntScan, NNSPLICE, GeneSplicer, and CADD). The predicted tertiary structure of the ABCA3 transporter showed the location of the missense variant p.M223T (Figure 3), and the variant was predicted to be deleterious by different algorithms (SIFT: 0.002, Damaging; Polyphen-2: 0.948, Probably_damaging; MutationTaster: 1.000, Disease_causing; FATHMM: 3.83, Damaging; and CADD: 23.4, Damaging).

According to the clinical phenotypes and genetic analysis, the patient was diagnosed as having ILD caused by bi-allelic mutations in the *ABCA3* gene. He was then treated with azithromycin 10 mg/kg/d for 3 days, dexamethasone 0.2 mg/kg/d for 5 days and 0.1 mg/kg/d for 18 days, and hydroxychloroquine 10 mg/kg/d for 20 days. However, the patient did not respond to these therapies and progressed into severe respiratory distress syndrome (RDS). Unfortunately, the patient died at 94 days of age.

#### Patient 2

Patient two was a 34-month-old boy who suffered from repeating cough from 9 months of age. The boy was delivered at full term (39 weeks), with a birth weight of 3,000 g. In the beginning the patient’s cough was mainly induced by cold temperature, exercise, and crying, which was accompanied by cyanotic complexion and wheezing. The cough still persisted after several outpatient and hospitalized treatments, which alleviated the symptoms after azithromycin treatment. Upon physical examination, the patient had pestle fingers. Chest X-ray revealed bilateral diffuse plaque-like shadows (Figure 1B). The chest HRCT scan showed multiple striped fuzzy shadows and ground-glass opacity in both lung fields (Figure 1B, 2a-c). His parents were both asymptomatic, and no family history of genetic disease was reported.

The genetic analysis showed compound heterozygous *ABCA3* variants; paternally inherited c.704T > C (p.F235S) in exon 8, and maternally inherited c.4037_4040del (p.T1346Nfs*15) in exon 27 (Figure 2B). The two variants in the *ABCA3* gene were both absent from the gnomAD and ExAC databases, and have not been reported in the literature. The predicted tertiary structure of the ABCA3 transporter showed the location of the missense variant p.M223T (Figure 3), and the variant was predicted to be deleterious by different algorithms (SIFT: 0.001, Damaging; Polyphen-2: 0.744, Probably_damaging; MutationTaster: 1.000, Disease_causing; FATHMM: 4.28, Damaging; and CADD: 21.0, Damaging).

Based on the clinical characteristics and genetic analysis, the patient was diagnosed to have ILD caused by *ABCA3* gene mutations. Subsequently, budesonide, ipratropium bromide atomization and acetylcysteine oral symptomatic treatments were given. The patient was slightly short of breath and had no obvious cyanosis. The patient was followed-up in the outpatient clinic after being discharged from hospital, and presented with alleviated clinical features without respiratory support at the age of 41 months.

## Whole Exome Sequencing

Whole exome sequencing and data analysis for the two patients and their patients were carried out in AmCare Genomic Laboratory (Guangzhou, China). Genomic DNA was extracted using the SolPure Blood DNA kit (Magen) according to manufacturer’s instructions and was fragmented by Q800R Sonicator (Qsonica). The paired-end libraries were prepared following Illumina library preparation the protocol. The whole-exomes were captured using the Agilent Sure-Select Human All Exon Kit (Agilent Technologies, Santa Clara, CA). Captured DNA samples were amplified by PCR with indexed primers and then sequenced on an Illumina NovaSeq 6,000 sequencer (Illumina, San Diego, CA). Raw-image data conversion and demultiplexing were performed following Illumina’s primary data analysis pipeline using CASAVA version 2.0 (Illumina). Low-quality reads (Phred score < Q20) were removed before demultiplexing. Sequences were aligned to the hg19 reference genome by NextGENe software (SoftGenetics, State College, PA) using the recommended standard settings for single-nucleotide variant and insertion/deletion discovery. The variants with minor allele frequency (MAF) > 1% in the Asian population and synonymous variants and were filtered out. Variant annotation was further confirmed through literature and population databases, including GnomAD (https://gnomad.broadinstitute.org/), Clinvar (https://www.ncbi.nlm.nih.gov/clinvar/), HGMD (http://www.hgmd.cf.ac.uk/ac/index.php), and OMIM (https://www.omim.org/). Genetic evaluation of pathogenicity of candidate gene variants was performed using multiple computational algorithms, including SIFT, Polyphen-2, MutationTaster, and CADD. The pathogenicity of the variants was estimated using the American College of Medical Genetics and Genomics (ACMG) guidelines (Richards et al., 2015).

## Discussion

Pediatric patients with ILD represent varying degrees of respiratory disorders with different age of onset and clinical manifestations. Chest HRCT scan is the key tool for the ILD diagnosis, while diffuse ground-glass attenuation is the most common feature. Once the diagnosis of ILD is made, the etiological diagnostic approach is required in patients. An underlying genetic causes occurs frequently in early onset ILD, and the genetic diagnosis is important for predicting prognosis and counseling families regarding recurrence risks. ILD manifesting in term newborns or young infants is usually due to mutations in the surfactant protein genes (Gupta and Zheng, 2017), or conditions related to congenital alveolar capillary dysplasia and congenital acinar dysplasia. In this study, we reported two unrelated pediatric patients with shortness of breath, dyspnea and hypoxemia, and chest HRCT findings of patchy ground-glass opacity in both lung fields, suggestive of diffuse ILD. Novel compound heterozygous variants in the *ABCA3* gene were identified using whole exome sequencing in the two patients. Patient one had paternally inherited c.4035+5G > A and c.668T > C (p.M223T), and maternally inherited c.1285+4A > C while patient two had c.704T > C (p.F235S) and c.4037_4040del (p.T1346Nfs*15).

The *ABCA3* gene is located on human chromosome 16p13.3 and contains 33 exons encoding a protein consisting of 1,704 amino acids. Protein Structure prediction algorithms shows that ABCA3 has a typical structure of most ABC transporters, consisting of two six-unit membrane-spanning domains, two nucleotide-binding domains (NBD1 and NBD2) oriented toward the cytosol, and six extracellular domains (ECD1-6) oriented towards the inner compartment of lamellar bodies (Peca et al., 2015). The ABCA3 glycoprotein uses the energy of ATP hydrolysis to translocate substrates across cell membranes. More specifically, ABCA3 transports surfactant phospholipids to lamellar bodies, which play a crucial role in maintaining the homeostasis of lung surfactant phospholipids (Schindlbeck et al., 2018). Bi-allelic mutations in the *ABCA3* gene were first reported in a group of full-term infants with severe neonatal surfactant deficiency occurring shortly after birth (Shulenin et al., 2004). ABCA3-related surfactant protein disorder is an autosomal recessive disease that leads to the dysfunction of phospholipid transporters related to lung surfactants. To date, more than 200 mutations in the *ABCA3* gene have been reported in patients with respiratory diseases, including nonsense, frameshift, spice-site, and missense mutations (Beers and Mulugeta, 2017; Wei et al., 2020; Bozkurt and Sahin, 2021; Zhang et al., 2021). Many deleterious mutations found in human tend to cluster on ECD1, ECD4, and NBD2 (Peca et al., 2015; Beers and Mulugeta, 2017). Experimental studies showed that ABCA3 proteins with ECD1 and ECD4 mutations remained localized in the endoplasmic reticulum and exhibited impaired glycosylation, whereas mutant proteins in NBD1 and NBD2 led to decreased ATP binding and/or hydrolysis (Matsumura et al., 2006; Wambach et al., 2016). In this study, the two novel missense variants c.668T > C (p.M223T) and c.704T > C (p.F235S) were located on ECD1, which were predicted to be deleterious by different algorithms.

Many evidences indicate that the *ABCA3* gene mutation-associated lung diseases are highly heterogeneous, ranging from RDS in term neonates to childhood ILD and fibrosing ILD in adults. A study of genotype-phenotype correlations in 185 infants and children with bi-allelic *ABCA3* mutations showed that all of the infants with frameshift and/or nonsense mutations presented with respiratory failure at birth compared with 75% of infants with other genotypes (non-allelic frameshift and/or nonsense mutations); all of the infants with frameshift and/or nonsense mutations have died or undergone lung transplantation by 1 year of age compared with 62% of infants/children with other genotypes (Wambach et al., 2014). A number of studies have shown that bi-allelic frameshift or nonsense *ABCA3* mutations are predictive of neonatal presentation and poor outcome, whereas the presentation and outcome for infants and children with other genotypes (missense, splice site, and in-frame mutations) were more variable and less predictable (Cho et al., 2020; Klay et al., 2020; Wang et al., 2021). Of note, many studies showed that monoallelic *ABCA3* mutations are also common in infants with RDS, childhood ILD, and adults with idiopathic pulmonary fibrosis (IPF) and diffuse parenchymal lung disease (DPLD) (Wambach et al., 2012; Coghlan et al., 2014). In this study, patient 1 with missense and intron compound heterozygous variants in the *ABCA3* gene presented with shortness of breath at birth, and then developed into severe RDS with a poor outcome. Moreover, patient 2 with a missense and a frameshift compound heterozygous variants presented with chronic repeated cough and hypoxemia at 9 months of age with a diagnosis of childhood ILD. He was given surface hormone inhalation therapy and is currently under follow-up.

Until now, there have been no specific therapeutic strategies for children with surfactant protein dysfunction caused by *ABCA3* mutations. In the acute setting, patients with increased respiratory effort and hypoxaemia could be provided with respiratory supportive care, ranging from oxygen supplementation to non-invasive or invasive ventilation. Most neonates presenting acute respiratory distress would initially receive surfactant replacement therapy, which may lead to a clinical response initially but not maintained in the longer term. Other drug treatments include corticosteroids, hydroxychloroquine, and azithromycin have been reported to be effective in some cases with *ABCA3* mutations (Hayes et al., 2012; Thouvenin et al., 2013; Nishida et al., 2021; Shaaban et al., 2021). Immunosuppression with corticosteroids are the preferred choice, administered intravenous methylprednisolone or oral prednisolone. Intravenous methylprednisolone is usually given at a dose of 10 mg/kg/day or 500 mg/m^2^ for 3 days consecutively at monthly intervals, assessing the clinical response to treatment after 7 days in children who are ventilated or at 28 days in non-ventilated children. Oral prednisolone is most commonly administered at a dose of 1 mg/kg/day, used in between pulses of methylprednisolone. Hydroxychloroquine can be started with a recommended dose of 6–10 mg/kg/day, and clinical response to treatment should be assessed after 3–4 weeks or after 3 months in children who are ventilated or not. Alternatively, azithromycin with a recommended dose of 10 mg/kg 3 days per week can be used as a second-line therapy (Bush et al., 2015). However, it is difficult to evaluate the effects of treatment with the above-mentioned drugs due to the lack of either randomized or controlled studies. Lung transplantation may be the only option for children with end-stage lung disease that is unresponsive to other therapies.

In conclusion, a diagnosis of surfactant protein dysfunction should be suspected in full-term newborns with unexplained respiratory distress and in older children with persistent unexplained respiratory symptoms such as cough, hypoxemia, and shortness of breath. The main HRCT characteristic manifestation is ground-glass opacification. Genetic testing now enables non-invasive diagnosis of surfactant protein dysfunction, negating the need for histology on lung biopsy. Timely genetic diagnosis is important for conducting management, predicting prognosis, and counseling families regarding recurrence risks. In this study, we reported the clinical and genetic features of two children with compound heterozygous variants in the *ABCA3* gene, and the novel mutations have expanded the spectrum of known mutations in the *ABCA3* gene. A deeper understanding of pathogenic mechanisms involved in ABCA3 deficiency may contribute to elucidate the genotype-phenotype correlations and the development of mutation specific drugs in the future.

## Data Availability

The datasets for this article are not publicly available due to concerns regarding participant/patient anonymity. Requests to access the datasets should be directed to the corresponding author.
